# Effects of Nonnutritive Sweeteners on Body Composition Changes during Pubertal Growth

**DOI:** 10.3390/nu15102319

**Published:** 2023-05-15

**Authors:** Yu-Hsin Chien, Chia-Yuan Lin, Shih-Yuan Hsu, Yue-Hwa Chen, Hung-Tsung Wu, Shiu-Wen Huang, Yang-Ching Chen

**Affiliations:** 1 Department of Education, Taipei Medical University Hospital, Taipei 110, Taiwan; 2 Department of Family Medicine, School of Medicine, College of Medicine, Taipei Medical University, Taipei 110, Taiwan; 3 Department of Food Science, National Taiwan Ocean University, Keelung City 202301, Taiwan; 4 School of Nutrition and Health Sciences, College of Nutrition, Taipei Medical University, Taipei 110, Taiwan; 5 Department of Internal Medicine, School of Medicine, College of Medicine, National Cheng Kung University, Tainan 701, Taiwan; 6 Graduate Institute of Medical Sciences, College of Medicine, Taipei Medical University, Taipei 110, Taiwan; 7 Department of Pharmacology, School of Medicine, College of Medicine, Taipei Medical University, Taipei 110, Taiwan; 8 Department of Medical Research, Research Center of Thoracic Medicine and Asthma, Taipei Medical University Hospital, Taipei 110, Taiwan; 9 Department of Family Medicine, Taipei Medical University Hospital, Taipei 110, Taiwan; 10 Graduate Institute of Metabolism and Obesity Sciences, Taipei Medical University, Taipei 110, Taiwan

**Keywords:** non-nutritive sweeteners, puberty, fat, fat-free mass, adiposity

## Abstract

The effects of consuming specific types of nonnutritive sweeteners (NNSs) on adiposity changes in children have remained inconsistent. In this study, we aimed to investigate the effects of the intake of different kinds of NNSs on long-term adiposity changes during pubertal growth. Furthermore, we examined the above relationships among different sexes, pubertal stages, and levels of obesity. A total of 1893 6–15-year-old adults were recruited and followed-up every 3 months. The NNS-FFQ (Food Frequency Questionnaire) was conducted and urine samples were collected to investigate the effects of the selected sweeteners, which included acesulfame potassium, aspartame, sucralose, glycyrrhizin, steviol glycosides, and sorbitol. Multivariate linear mixed-effects models were used to examine the relationship between NNS intake and body composition. The consumption of aspartame, sucralose, glycyrrhizin, stevioside, and sorbitol was associated with decreased fat mass and increased fat-free mass. In the highest tertile group, the effects of NNS consumption on fat mass corresponded to values of −1.21 (95% CI: −2.04 to −0.38) for aspartame, −0.62 (95% CI: −1.42 to 0.19) for sucralose, −1.26 (95% CI: −2.05 to −0.47) for glycyrrhizin, −0.90 (95% CI: −2.28 to 0.48) for stevioside, and −0.87 (95% CI: −1.67 to −0.08) for sorbitol, while the effects on fat-free mass corresponded to values of 1.20 (95% CI: 0.36 to −0.38) for aspartame, 0.62 (95% CI: −0.19 to 1.43) for sucralose, 1.27 (95% CI: 0.48 to 2.06) for glycyrrhizin, 0.85 (95% CI: −0.53 to 2.23) for stevioside, and 0.87 (95% CI: 0.08 to 1.67) for sorbitol. Particularly, aspartame and sorbitol revealed a dose-responsiveness effect. The above finding was more prominent among girls than boys. Moreover, fat mass was significantly reduced in normal-weight children who consumed a moderate amount of aspartame and a large amount of glycyrrhizin and sorbitol compared with obese children. In conclusion, the NNS-specific and sex-specific effects of long-term NNS consumption revealed associations of decreasing fat mass and increasing fat-free mass for children undergoing pubertal growth.

## 1. Introduction

Obesity in children and adolescents is an increasingly concerning global health issue. In 2016, more than 340 million children and adolescents aged 5–19 worldwide were estimated to be overweight or obese [1]. The 2016 Health Survey of the Ministry of Education in Taiwan indicated that 14.9% of elementary school students and 16.8% of junior high school students were obese [2]. The risk factors for obesity include genetics, environmental conditions, and dietary practices [3]. In particular, added sugar intake is strongly associated with obesity, and this has prompted the population-wide recommendation that sugar consumption should be reduced [4]. The World Health Organization (WHO) strongly recommends that the share of sugar in a person’s total energy intake should be <10%, preferably as low as 5% [5]. Sugar replacements or nonnutritive sweeteners (NNSs) have thus gained enormous popularity owing to their low caloric value and perceived health benefits [6].

The increase in the consumption of food and beverages containing NNSs has raised concerns about the potential adverse health effects of these substances [7,8,9]. Studies have investigated their short-term consequences (e.g., for food intake, mood, blood pressure, and blood glucose) and long-term health effects (e.g., on body weight; incidence of obesity; and risk of cancer, diabetes, and dental caries). Various beneficial and adverse health effects of NNSs have been postulated. A comprehensive review established a relationship in children and adolescents between NNS consumption and weight gain [10]. Many cross-sectional observational studies and prospective cohort studies have indicated a positive association between NNS intake and body mass index (BMI) change [11,12,13,14,15], whereas some have not indicated such an association [16,17]. Due to the inconsistent results reported from randomized controlled trials [18,19,20,21,22], further longitudinal studies are warranted.

Notably, some studies have discovered different odds of obesity among boys and girls. A 2007 prospective cohort study [11] and a 2017 cross-sectional study [15] revealed a positive association between diet soda intake and BMI gain in boys, whereas a 2016 cross-sectional study [14] indicated positive dose-dependent associations of diet soda intake with BMI, body fat, and odds of obesity in girls. Moreover, sex-specific NNS effects were observed in prenatal NNS exposure [23,24] and rodent models [25,26,27]. Overall, the sex-specific effects of NNSs on adiposity remain unclear.

Our study addressed the limitations of existing studies. First, our existing results must be interpreted with caution due to the potential for confounders, including recall bias, given that scholars have relied on participants to identify and report the type and number of NNS-containing products they consumed. Biomarkers can quantitatively assess NNS intake. Second, due to the differences in chemical structure between different types or sources of NNSs, NNSs should be distinguished to prevent multifaceted interventions, which would make it impossible to isolate the specific effects of NNSs. Third, few studies have been conducted in countries other than the United States, Canada, and the United Kingdom, which has affected the global generalizability of current findings. Therefore, studies conducted in different populations such as the Asian population are required. 

In the present study, we investigated the effect of the consumption of various types of NNSs on long-term body composition during pubertal growth. We examined the correlations between urinary NNS levels and body composition through a sensitivity analysis. We explored the aforementioned association among groups of different sexes, pubertal stages, and levels of obesity [28]. 

## 2. Materials and Methods

### 2.1. Study Design and Data Collection

**Figure 1:**
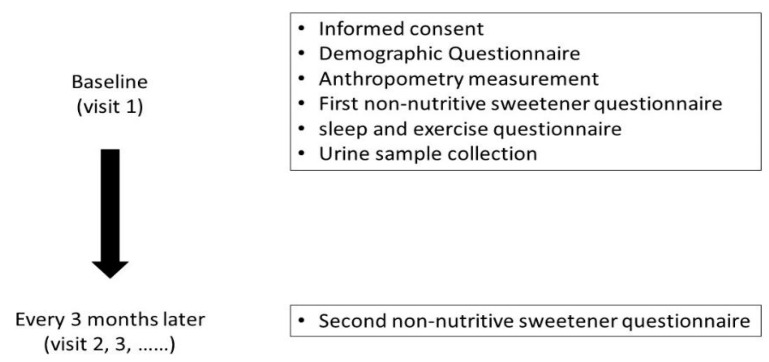
Study design and the timeline for each measurement.

The Taiwan Puberty Longitudinal Study (TPLS) is a multidisciplinary longitudinal study of children undergoing puberty. A total of 1893 eligible adolescents aged 6–15 were recruited from pubertal and pediatric endocrine outpatient clinics in multiple centers in Taiwan, namely, Taipei Medical University Hospital, Taipei Municipal Wanfang Hospital, Cathay General Hospital in Taipei, and National Cheng Kung University Hospital, from 2018 to 2021. This study excludes subjects with metabolic disorders and congenital conditions, such as diabetes, hyperlipidemia, maple syrup urine disease, and phenylketonuria. (Figure 1). They were followed-up every 3 months until the end of puberty. The average length of the follow-up interval was 3.45 months. Demographic background information, dietary records, Non-Nutritive Sweetener Food Frequency Questionnaire (NNS-FFQ) scores, and spot urine data were collected during recruitment for the study. As most of the adolescents were unwilling to collect their urine data, there were fewer children with urine samples than would be typically represented in dietary recall assessments. This study was approved by the Institutional Review Board of Taipei Medical University (N202003013), Cathay General Hospital (CGH-P108107), and National Cheng Kung University Hospital (B-BR-108-076) and complied with the principles outlined in the Declaration of Helsinki. All the participants provided informed consent for this study.

### 2.2. Exposure Assessment

A pediatric endocrinologist assessed breast development and pubic hair development for each girl. The pubertal stages in breast and pubic hair development were graded in accordance with the 5-stage scale described by Marshall and Tanner [29]. When the 2 breasts of an individual were not at the same stage of development, the stage of the more advanced side was recorded. A pediatric endocrinologist assessed the male adolescents’ testicular volume through comparative palpation with a Prader orchidometer [30]. If the testes of an individual were not identical, the volume of the larger one was recorded.

Demographic background information—sex, age, race, anthropometric data, education level, and household income—was recorded at recruitment. Body height and weight were measured to the nearest 0.1 cm and 0.1 kg, respectively. BMI was calculated and expressed in kg/m^2^. Cutoff points of age-specific and sex-specific 85th and 95th percentiles were used to define overweight and obesity, respectively, according to the Growth Charts for Taiwanese Children [31]. A 24 h dietary record of total energy intake was obtained by a trained registered dietitian. Energy and food intake was estimated using the Nutritionist edition, COFIT Pro, Version 1.0.0, a software package for nutrient analysis that uses a Taiwanese food composition table as a nutrient database [32]. The types and amounts of NNSs consumed by the adolescents were determined using a database for NNS-containing food reported by manufacturers. Products that did not reveal a clear concentration of NNSs were sent to SGS Taiwan Limited, Nankang, for analysis of their NNS concentrations. The online FFQ was reviewed and completed by the participants.

### 2.3. Establishment of the Semiquantitative NNS-FFQ

We conducted intensive and wide-ranging market research to establish the comprehensive semiquantitative NNS-FFQ in our previous study [28], which estimates a child’s NNS consumption level in the 3 months preceding the time of examination. While establishing the FFQ, common food products containing one or more types of NNS were identified. The brand name and flavor of each food product were filed, photo-recorded, and then classified into 13 categories; consequently, 305 food items are present in the questionnaire. The intake of six intense sweeteners—acesulfame potassium, aspartame, sucralose, glycyrrhizin, steviol glycosides, and sorbitol—was calculated by multiplying the concentration by portion size and frequency. The intake of added sugars—raw sugar, dextrose, fructose, sucrose, glucose, lactose, high-fructose corn syrup, and honey—was estimated with reference to the FFQ food items. The examined NNSs were mostly present in diet drinks and low-calorie drinks. Our previous study [28] discovered significant moderate correlations between estimated steviol glycoside or sucralose consumption and sensitive urinary biomarker levels (κ = 0.59 and 0.45, respectively).

A total of 455 children within the TPLS cohort whose urine samples were available were included in the present analyses. The levels of acesulfame potassium, sucralose, and steviol glycosides in urine were examined using liquid chromatography–mass spectrometry through an experimental protocol described in a previous study [28]. The concentration of each NNS excreted in urine was calibrated using the creatinine level and then expressed as μg/g Cr [28].

Weight and body composition (fat mass, fat-free mass, protein weight, and water weight) were measured when the participant was wearing light clothing and by using a portable bioimpedance analysis electronic scale TT-BC418 (Tannita, Japan) [33]. BMI was converted into a BMI z-score by consulting WHO Growth Standards. Waist and hip circumferences were measured to the nearest 1 mm using flexible tape. Skinfold thickness was measured in duplicate to the closest 0.5 mm with Lange calipers (Beta Technology, Santa Cruz, CA, USA) at the bilateral triceps and gastrocnemius.

### 2.4. Covariate Assessment

Confounders in the statistical models were a priori confounders based on previous research with data relevant to both puberty outcomes and NNSs. All models were adjusted for sex, age, amount of exercise, sleep quality, total energy intake, and parental education time. Amount of exercise was defined in accordance with the International Physical Activity Questionnaire (IPAQ) and divided into three categories: mild (<3 days of exercise per week), moderate (3–6 days of exercise per week), and vigorous (>6 days of exercise per week). Sleep quality was defined in accordance with the Pittsburgh Sleep Quality Index questionnaire; index values were used to divide the children into those with poor versus good sleep quality.

### 2.5. Statistical Analysis

We compared the variables that potentially affect adolescents’ body composition (Table 1). The *t*-test and chi-square test were employed for continuous and categorical variables, respectively, to compare the demographic data between boys and girls. Multivariate regression and linear mixed-effects modeling (LMER) [34] were used to examine the relationship between children’s average daily NNS intake and body composition every 3 months. LMER was used to analyze repeated measurement data, accounting for the correlations between different observations of the same person at different times, thereby capturing the longitudinal effect of NNS consumption on body composition. Models were adjusted for variables that may affect children’s body composition, including parental education time, family income, sleep quality, amount of exercise, and total energy intake. We divided the adolescents into groups based on consumption tertiles (T1–T3) by considering their proportion of daily intake to acceptable daily intake (ADI) [35] and compared the body compositions of NNS-consuming groups to those of the no-consumption group. If the dietary intakes for the sweeteners were estimated several times due to there being several FFQ records, we would use the first record as the baseline data for defining tertiles.

The mice package in R version 4.0.3 was used to fill in missing values of continuous variables, whereas the mode was used to fill in the missing values of categorical variables. All tests were two-sided, for which a 5% significance level was set. All analyses were performed using R version 4.0.3 (R Foundation for Statistical Computing, Vienna, Austria). The corresponding packages used were lmerTest and lme4.

## 3. Results

### 3.1. Demographic Characteristics

The study population comprised 1239 girls and 654 boys (mean age: 9.69 and 11.78 years, respectively). Their total calorie intake values were 1494.79 and 1792.37 kcal, respectively. Acesulfame potassium, aspartame, and sucralose intake levels were significantly higher among boys than among girls (all *p* < 0.05). The percentage of fat mass was higher among girls than among boys (Table 1 and Table S2).

### 3.2. Association between NNS Consumption and Body Composition

**Figure 2:**
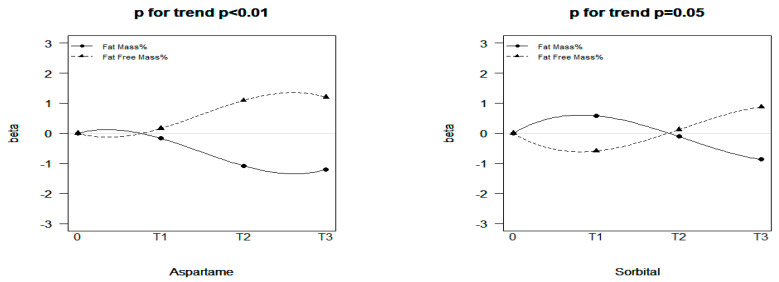
Dose—response effects of aspartame and sorbitol consumption on fat mass and fat-free mass.

Aspartame, sucralose, glycyrrhizin, stevioside, and sorbitol consumption in the T2 and T3 groups was associated with decreased fat mass and increased fat-free mass (Table 2). Consumption of added sugars was associated with increased fat mass and decreased fat-free mass (T1 group). For the T2 and T3 groups, the added sugar group also showed a sharp decrease in fat mass and an increase in fat-free mass like the other NNS groups; however, the statistics were not significant. A significant dose–response effect was observed for aspartame and sorbitol (Figure 2). Consumption of aspartame in groups T1 to T3 had a strengthening negative effect on fat mass, with the beta values equal to −0.17 (95% CI: −0.98 to 0.63), −1.08 (95% CI: −1.95 to −0.21), and −1.21 (95% CI: −2.04 to −0.38), respectively. Moreover, the fat-mass-reducing effect of sorbitol from T1 to T3 also exhibited dose-responsiveness, with the beta values equaling 0.58 (95% CI: −0.33 to 1.50), −0.11 (95% CI: −0.89 to 0.67), and −0.87 (95% CI: −1.67 to −0.08), respectively. The correlations between urinary NNS levels and body composition obtained in the sensitivity analysis are presented in Table S1. In general, urinary NNS concentration still exhibited an effect of a decrease in fat mass and increase in fat-free mass. Specifically, in group T1, sucralose had a significant effect on the decrease in fat mass (β: −3.00; 95% CI: −5.59 to −0.41) and increase in fat-free mass (β: 3.02; 95% CI: −0.43 to 5.61; Table S1).

### 3.3. Association between NNSs and Body Composition in Different Sexes

Regarding intake of acesulfame potassium, aspartame, sucralose, and glycyrrhizin, girls exhibited a greater tendency than boys to undergo a decrease in fat mass and an increase in fat-free mass. We observed the different effects of NNS consumption on body composition according to sex (Table 3). In group T3, stevioside consumption was significantly different between boys and girls (*p* = 0.02). The beta value for the association of stevioside with fat mass was −3.7 (95% CI: −6.66 to −0.74), while that for the association with fat-free mass was 3.65 (95% CI: 0.69–6.61) in boys, constituting the most notable statistical changes among all groups. In girls, increased fat mass and decreased fat-free mass were observed in group T2. For the T2 and T3 groups, the added sugar group also showed a decrease in fat mass and an increase in fat-free mass like the other NNS groups, but the statistics were not significant.

### 3.4. Association between NNSs and Body Composition in Different Tanner Stages

We did not observe a significant difference in the fat-mass-reducing effect in the early or late stages of pubertal growth (Table 4). In the late stage of pubertal growth (Stages III–V), the effect of increasing fat mass and decreasing fat-free mass seemed to be stronger, as revealed by the beta value. For the T2 and T3 groups, the added sugar group showed a decrease in fat mass and an increase in fat-free mass; however, the statistics were not significant. Tables S3 and S4 present the results of the pubertal growth of girls and boys, respectively. The effect of increasing fat mass and decreasing fat-free mass seemed to be stronger in both sexes for high amounts of aspartame and glycyrrhizin. For high amounts of sorbitol, the aforementioned effect was only observed for boys.

### 3.5. Association between NNSs and Body Composition in Obese or Normal-Weight Children

The fat-mass-reducing effect was significant (Table S5) among normal-weight children consuming a moderate amount of aspartame (β: −0.92; 95% CI: −1.72 to −0.12), a high amount of glycyrrhizin (β: −1.18; 95% CI: −1.91 to −0.45), or a high amount of sorbitol (β: −0.88; 95% CI: −1.61 to −0.15), as was the fat-free-mass-increasing effect among normal-weight children consuming a moderate amount of aspartame (β: 0.93; 95% CI: 0.13–1.73), a high amount of glycyrrhizin (β: 1.18; 95% CI: 0.46–1.91), or a high amount of sorbitol (β: 0.88; 95% CI: 0.15–1.61).

## 4. Discussion

This longitudinal analysis has several novel findings. Aspartame, sucralose, glycyrrhizin, stevioside, and sorbitol consumption was associated with decreases in fat mass and increases in fat-free mass, with a dose–response effect discovered for aspartame and sorbitol. Moreover, a sex-specific effect existed in these associations. Regarding the consumption of acesulfame potassium, aspartame, sucralose, and glycyrrhizin, girls exhibited a greater tendency than boys to have a decrease in their fat mass and increase in their fat-free mass; in boys, this tendency was marked for stevioside. The fat-mass-reducing effect was more significant for normal-weight children who consumed a moderate amount of aspartame, a large amount of glycyrrhizin, or sorbitol than in their obese counterparts. Our findings indicate the NNS-specific and sex-specific effects of long-term NNS consumption on body composition for children undergoing pubertal growth. Further large-sample, randomized controlled trials are warranted to provide clinical recommendations.

The impacts of chronic NNS consumption on the risk and development of obesity and metabolic syndrome remain controversial. Few studies have investigated metabolic outcomes among children and adolescents who consume NNSs. The limited observational studies conducted by pediatric patients have suggested that there is a positive association between increased BMI in children who consume NNS-sweetened carbonated soft drinks [13,36,37], increased body fat accumulation [12,13,38,39], and obesity [40]. However, because these studies failed to clearly distinguish specific types of NNS to avoid multifaceted interventions, their findings may be inconsistent with our results. In their rodent study, Palmnäs et al. [41] reported that chronic consumption of a small amount of aspartame in the water of a diet-induced-obesity rat model resulted in lower body mass than the equivalent consumption of sugar-sweetened water. The aspartame-exposed groups consumed fewer net calories despite having a diet with an identical composition to other groups because aspartame was only administered in drinking water. However, these groups presented fasting hyperglycemia and impaired insulin tolerance, which might have been due to enhanced gluconeogenesis fueled by propionate production caused by compositional changes in gut microbiota. Pandurangan et al. [42] discovered that aspartame also significantly reduced lipid accumulation and the expression of peroxisome proliferator-activated receptor γ, fatty-acid-binding protein 4, CCAAT/enhancer-binding protein α, and sterol-regulatory-element-binding protein 1 during 3T3-L1 differentiation without having a significant toxic effect. Glycyrrhizic acid prevents increases in adipocyte size, triglycerides, and circulating leptin levels [43,44,45,46]. The decrease in visceral adipocyte size with glycyrrhizin intake was attributed to decreased circulating insulin levels and increased lipoprotein lipase expression in multiple tissues. Such a change could lead to a competitively increased uptake of free fatty acids into non-adipose tissues and a consequentially smaller amount of circulating lipids available for accumulation in adipose tissue. Masubuchi et al. [47] reported reduced adipogenesis in 3T3-L1 cells upon stimulation with sucralose (20 mM) due to the activation of the adenylate cyclase–cAMP signaling pathway. In their randomized controlled trial, Higgins et al. [48] compared the effects on body weight of different NNSs. The body weight of those consuming sucralose tended to decrease and was significantly lower than that of those consuming any other sweetener. They suggested that decreased energy intake and oral intake frequency with sucralose consumption corresponded to weight reduction.

Few studies have analyzed why girls tend to exhibit a greater decrease in fat mass and increase in fat-free mass. During puberty, luteinizing hormone and follicle-stimulating hormone activate maturational changes in the gonads. The maturing ovaries and testes secrete the gonadal steroids estrogen and testosterone, respectively [45]. Estrogen can promote the accumulation of subcutaneous fat [46], which contributes to the generally higher amount of body fat observed in girls. Palmnas and colleagues [41] concluded that aspartame reduced body fat percentage and plasma insulin levels in individuals on a high-fat diet incorporating aspartame compared with a standard chow diet incorporating an identical amount of aspartame. We speculate that the greater increase in body fat among girls during puberty may explain their stronger tendency for fat mass reduction.

The fat mass reduction effect was stronger among normal-weight children than obese children, even after adjustment for total energy intake. Palmnas and colleagues [41] also concluded that aspartame was associated with an increased proportion of Enterobacteriaceae when combined with a high-fat diet. Members of the Proteobacteria phylum, including Enterobacteriaceae, produce gases and short-chain fatty acids that are associated with inflammation and insulin resistance. We propose that children with a normal BMI have a greater fat-mass-reducing tendency because of their healthier eating habits and, consequently, lower levels of fat consumption, whereas obese children may consume more fat, leading to increased inflammation and insulin resistance, thereby attenuating the fat-mass-reducing effect.

Our study has several strengths. First, we conducted a detailed market survey to differentiate several types of NNSs to avoid a general assessment of artificially sweetened beverages. This was a key breakthrough compared with most previous observational studies in which the separate effects of different NNSs were not calculated. Second, we assessed urine test results to more precisely analyze NNS intake. Despite the small sample for the urinary NNS analysis, we discovered a similar effect of fat mass reduction under NNS exposure. Urinary NNS measurement also compensated for the recall bias that was introduced because the NNS-FFQ was used. Third, we performed subgroup analyses according to sex and Tanner stage. During puberty, several maturational changes occur in physiology and body composition. Since different people mature at different times, we carefully categorized adolescents into Tanner stages. No human or rodent studies have reached a definitive conclusion related to the sex-specific effects of NNS intake; ours is the first study to conduct detailed statistical analysis of both sexes. Finally, we closely followed up with the participants every 3 months, which enabled us to record and analyze the within-individual variation in fat mass growth.

Our study has some limitations. Its observational design precluded determinations of causality. However, repeated measurements within the LMER model were used, mimicking the effect of temporal causality. Recall bias related to the NNS drink consumption level might have influenced the results, but our previous study (Public Health Nutrition, under revision) proved the reliability and validity of our NNS-FFQ. On the other hand, we observed a similar trend of the effect of added sugar on body composition as compared to that observed with NNSs, although the results were non-significant. We suggest that large, prospective, interventional children-focused studies should be conducted to investigate and compare the effects of NNSs and added sugar on adiposity growth.

## 5. Conclusions

Our study offers a reference for future observational or randomized controlled trials regarding the selection of specific types of NNS in research. Although the results of decreasing fat mass and increasing fat-free mass symbolize a positive change in body composition, studies concerning NNSs have revealed insulin tolerance [41,49,50,51,52,53,54] and gut microbiota change [41,53,54] in people who consume NNSs, which remain controversial issues that have not been solved. As NNS exposure usually begins in early childhood, prospective studies must be conducted to determine whether chronic NNS consumption throughout childhood increases the risk of obesity or metabolic disease, and physiological changes due to NNS intake must also be clarified. Animal models of NNS consumption are critical for testing hypotheses and determining the biological mechanisms that drive the findings in epidemiological studies. Future longitudinal studies and animal models should be conducted to determine the long-term effects of NNSs and whether they should be recommended for children and adolescents and whether the potential weight decrease benefits are acceptable given the negative metabolic outcomes during this critical developmental period. Future studies should attempt to establish clear guidelines on NNS-specific safety and usage.

## Figures and Tables

**Table 1:** Baseline characteristics of the participants.

Characteristics	Girls		Boys		*p* Value
*n*	1239		654		
Age (years)	9.69	1.82 (SD)	11.78	1.93 (SD)	<0.01
Birth weight (g)	2922.14	563.43 (SD)	3012.23	532.96 (SD)	<0.01
Breastfed	857	83.69%	415	80.74%	0.17
Poor sleep quality	219	20.64%	139	25.93%	0.02
Total calorie intake (kcal)	1494.79	426.99 (SD)	1792.37	462.59 (SD)	<0.01
Parental education					0.36
Senior high school or below	82	7.97%	34	6.63%	
College	591	57.43%	285	55.56%	
Graduate school or higher	356	34.60%	194	37.82%	
Family income NTD					0.03
<70,000	119	11.69%	39	7.68%	
70,000–100,000	374	36.74%	182	35.83%	
>100,000	525	51.57%	287	56.50%	
Physical activity METD					0.03
Mild (<3 kcal/kg/h)	345	55.83%	100	29.33%	
Moderate (3~6 kcal/kg/h)	147	23.79%	90	26.39%	
Vigorous (>6 kcal/kg/h)	126	20.39%	151	44.28%	
Tanner stage					0.02
Tanner I and II	647	77.67%	394	83.12%	
Tanner III–V	186	22.33%	80	16.88%	
NNS consumption					
Acesulfame-K	198	21.50%	155	33.99%	<0.01
Aspartame	314	34.09%	198	43.42%	<0.01
Sucralose	348	37.79%	201	44.08%	0.03
Glycyrrhizin	322	34.96%	144	31.58%	0.24
Stevioside	119	12.92%	70	15.35%	0.25
Sorbitol	477	51.79%	212	46.49%	0.07
Added sugar	615	66.78%	300	65.79%	0.78
Absolute intake (mg)/(%ADI)	(mg)	(%ADI)	(mg)	(%ADI)	
Acesulfame-K	0.0007	0.0040	0.0023	0.0094	<0.01
Aspartame	0.0004	0.0021	0.0010	0.0050	0.01
Sucralose	0.0021	0.0091	0.0040	0.0129	0.02
Glycyrrhizin	0.0009	0.0029	0.0007	0.0023	0.06
Stevioside	0.0010	0.0040	0.0023	0.0094	0.11
Sorbitol	0.0003	0.0008	0.0004	0.0015	0.48
Added sugar	0.0037	0.0076	0.0036	0.0065	0.65
Body mass index kg/m^2^ (*z*-score)	0.19	1.34	0.39	1.47	<0.01
Obesity	140	11.41%	86	13.35%	0.25
Fat mass (%)	20.13	8.49	18.66	11.66	0.01
Fat-free mass (%)	79.87	8.48	81.34	11.67	0.01
Waist circumference (cm)	60.14	21.58	66.87	30.06	<0.01
Waist-to-hip ratio (%)	0.84	0.33	0.85	0.33	0.36
Waist-to-height ratio (%)	0.44	0.15	0.46	0.93	0.06

Definition of abbreviations: ADI = acceptable daily intake; METD = daily metabolic equivalent value; NTD = New Taiwan Dollar. Children with age- and sex-specific BMI under the 5th percentile, between the 5th and 85th percentile, between the 85th and 95th percentile, and over the 95th percentile were defined as underweight, normal weight, overweight, and obese, respectively, in accordance with the standards established by the WHO. Poor sleep quality—a Pittsburgh Sleep Quality Index (PSQI) score of ≥7; Tanner stage—pediatric endocrinologists evaluated the Tanner stage at outpatient clinic every visit.

**Table 2:** Associations between sweetener consumption and body composition (Taiwan Pubertal Longitudinal Study).

Sweetener	Exposure Amount *	*n*	BMI-z Score				Fat Mass %				Fat-Free Mass %				Waist-to-Height Ratio			
			β	95% CI		*p* Value	β	95% CI		*p* Value	β	95% CI		*p* Value	β	95% CI		*p* Value
Acesulfame-K	0	944	ref	-	-	-	ref	-	-	-	ref	-	-	-	ref	-	-	-
	T1	109	−0.05	−0.19	0.09	0.48	0.25	−0.89	1.39	0.67	−0.25	−1.39	0.89	0.67	<0.01	−0.02	0.02	0.84
	T2	112	−0.17	−0.30	−0.04	0.01	−1.02	−2.06	0.03	0.06	1.02	−0.03	2.07	0.06	−0.01	−0.03	0.01	0.40
	T3	115	−0.11	−0.24	0.02	0.09	−0.11	−1.16	0.94	0.84	0.10	−0.95	1.15	0.86	−0.02	−0.04	<0.01	0.13
Aspartame	0	816	ref	-	-	-	ref	-	-	-	ref	-	-	-	ref	-	-	-
	T1	157	0.01	−0.09	0.10	0.91	−0.17	−0.98	0.63	0.68	0.16	−0.65	0.97	0.70	<0.01	−0.02	0.02	0.75
	T2	167	−0.20	−0.31	−0.10	<0.01	−1.08	−1.95	−0.21	0.02	1.09	0.22	1.97	0.01	0.01	−0.01	0.03	0.42
	T3	166	−0.16	−0.27	−0.06	<0.01	−1.21	−2.04	−0.38	<0.01	1.20	0.36	2.03	0.01	0.01	−0.01	0.03	0.30
Sucralose	0	778	ref	-	-	-	ref	-	-	-	ref	-	-	-	ref	-	-	-
	T1	183	−0.06	−0.15	0.04	0.27	0.05	−0.75	0.85	0.90	−0.03	−0.83	0.78	0.95	0.01	−0.01	0.03	0.25
	T2	173	−0.13	−0.24	−0.03	0.01	−1.20	−2.02	−0.37	<0.01	1.18	0.36	2.01	<0.01	<0.01	−0.01	0.02	0.69
	T3	174	−0.14	−0.24	−0.04	0.01	−0.62	−1.42	0.19	0.13	0.62	−0.19	1.43	0.13	−0.01	−0.03	<0.01	0.11
Glycyrrhizin	0	860	ref	-	-	-	ref	-	-	-	ref	-	-	-	ref	-	-	-
	T1	147	−0.04	−0.14	0.06	0.44	−0.81	−1.64	0.01	0.05	0.82	<0.01	1.64	0.05	<0.01	−0.02	0.02	1.00
	T2	145	−0.03	−0.13	0.06	0.50	−0.28	−1.07	0.51	0.48	0.30	−0.49	1.10	0.45	<0.01	−0.02	0.02	0.89
	T3	164	−0.18	−0.27	−0.08	<0.01	−1.26	−2.05	−0.47	<0.01	1.27	0.48	2.06	<0.01	−0.01	−0.03	0.01	0.23
Stevioside	0	1088	ref	-	-	-	ref	-	-	-	ref	-	-	-	ref	-	-	-
	T1	63	0.05	−0.10	0.19	0.53	0.14	−1.04	1.32	0.82	−0.18	−1.36	1.00	0.77	<0.01	−0.03	0.03	0.92
	T2	61	−0.18	−0.34	−0.03	0.02	−1.24	−2.49	<0.01	0.05	1.26	0.01	2.50	0.05	0.01	−0.02	0.04	0.40
	T3	62	−0.06	−0.24	0.11	0.46	−0.90	−2.28	0.48	0.20	0.85	−0.53	2.23	0.23	−0.01	−0.03	0.02	0.69
Sorbitol	0	661	ref	-	-	-	ref	-	-	-	ref	-	-	-	ref	-	-	-
	T1	199	0.08	−0.04	0.19	0.18	0.58	−0.33	1.50	0.21	−0.59	−1.50	0.33	0.21	0.01	−0.01	0.03	0.17
	T2	227	0.01	−0.09	0.10	0.92	−0.11	−0.89	0.67	0.78	0.12	−0.66	0.91	0.76	<0.01	−0.01	0.02	0.66
	T3	222	−0.12	−0.21	−0.02	0.02	−0.87	−1.67	−0.08	0.03	0.87	0.08	1.67	0.03	−0.01	−0.03	0.01	0.26
Added sugar	0	453	ref	-	-	-	ref	-	-	-	ref	-	-	-	ref	-	-	-
	T1	289	0.07	−0.01	0.16	0.11	0.69	−0.01	1.38	0.05	−0.68	−1.38	0.02	0.06	0.01	−0.01	0.03	0.20
	T2	286	−0.06	−0.14	0.03	0.22	−0.23	−0.94	0.49	0.54	0.24	−0.48	0.96	0.51	<0.01	−0.01	0.02	0.65
	T3	303	−0.08	−0.17	0.01	0.09	−0.26	−1.01	0.49	0.50	0.28	−0.47	1.03	0.47	−0.01	−0.02	0.01	0.54

* Exposure amount was estimated as the proportion of daily intake with respect to ADI and was categorized into tertiles (T1–T3), with the no-intake group serving as a reference. The models were adjusted for age, sex, amount of exercise, sleep quality, total energy intake, and parental education level.

**Table 3:** Associations of sweetener consumption with fat mass and fat free mass according to sex.

Sweetener	Exposure Amount *	*n*			Fat Mass %									Fat-Free Mass %								
			*n*	*n*	Girls				Boys				*p* **	Girls				Boys				*p* **
			Girls	Boys	β	95% CI		*p*	β	95% CI		*p*		β	95% CI		*p*	β	95% CI		*p*	
Acesulfame-K	0	936	258	678	ref	-	-	-	ref	-	-	-	-	ref	-	-	-	ref	-	-	-	-
	T1	143	47	96	0.46	−0.79	1.71	0.47	−0.41	−2.66	1.84	0.72	0.30	−0.47	−1.72	0.78	0.46	0.41	−1.84	2.67	0.72	0.29
	T2	135	53	82	−1.21	−2.31	−0.10	0.03	−1.20	−3.39	0.98	0.28	0.67	1.21	0.11	2.32	0.03	1.19	−1.00	3.38	0.29	0.68
	T3	127	67	60	−1.20	−2.54	0.14	0.08	0.34	−1.37	2.05	0.70	0.25	1.17	−0.17	2.51	0.09	−0.34	−2.05	1.37	0.70	0.27
Aspartame	0	837	235	602	ref	-	-	-	ref	-	-	-	-	ref	-	-	-	ref	-	-	-	-
	T1	213	54	159	−0.71	−1.54	0.12	0.09	0.60	−1.29	2.49	0.54	0.10	0.68	−0.15	1.51	0.11	−0.57	−2.46	1.33	0.56	0.12
	T2	192	77	115	−1.33	−2.30	−0.37	0.01	−1.14	−2.82	0.55	0.19	0.72	1.36	0.39	2.32	0.01	1.13	−0.56	2.81	0.19	0.70
	T3	195	85	110	−1.45	−2.42	−0.47	<0.01	−1.27	−2.75	0.22	0.10	1.00	1.42	0.45	2.40	<0.01	1.26	−0.23	2.75	0.10	0.98
Sucralose	0	804	231	573	ref	-	-	-	ref	-	-	-	-	ref	-	-	-	ref	-	-	-	-
	T1	224	69	155	−0.08	−1.00	0.85	0.87	−0.53	−2.02	0.96	0.49	0.60	0.10	−0.83	1.03	0.83	0.56	−0.93	2.05	0.46	0.59
	T2	204	70	134	−0.83	−1.79	0.13	0.09	−1.88	−3.34	−0.41	0.01	0.36	0.82	−0.14	1.79	0.09	1.86	0.39	3.33	0.01	0.37
	T3	196	78	118	−1.16	−2.08	−0.23	0.01	−0.12	−1.64	1.40	0.87	0.18	1.16	0.24	2.09	0.01	0.13	−1.39	1.65	0.87	0.19
Glycyrrhizin	0	867	269	598	ref	-	-	-	ref	-	-	-	-	ref	-	-	-	ref	-	-	-	-
	T1	184	53	131	−0.59	−1.39	0.21	0.15	−1.54	−3.77	0.70	0.18	0.62	0.61	−0.19	1.41	0.14	1.53	−0.71	3.77	0.18	0.64
	T2	190	52	138	−0.58	−1.38	0.22	0.16	−0.06	−1.98	1.87	0.95	0.32	0.62	−0.18	1.42	0.13	0.04	−1.89	1.97	0.97	0.29
	T3	189	55	134	−1.00	−1.85	−0.14	0.02	−1.73	−3.31	−0.16	0.03	0.83	1.03	0.17	1.88	0.02	1.70	0.12	3.28	0.03	0.77
Stevioside	0	1041	293	748	ref	-	-	-	ref	-	-	-	-	ref	-	-	-	ref	-	-	-	-
	T1	79	28	51	−0.14	−1.41	1.13	0.83	1.20	−1.28	3.68	0.34	0.68	0.09	−1.18	1.36	0.89	−1.23	−3.71	1.25	0.33	0.69
	T2	70	31	39	−1.52	−2.91	−0.13	0.03	−0.55	−2.90	1.80	0.65	0.40	1.50	0.11	2.89	0.04	0.62	−1.74	2.98	0.61	0.44
	T3	71	23	48	0.07	−1.40	1.54	0.93	−3.70	−6.66	−0.74	0.01	0.02	−0.12	−1.59	1.35	0.87	3.65	0.69	6.61	0.02	0.02
Sorbitol	0	714	229	485	ref	-	-	-	ref	-	-	-	-	ref	-	-	-	ref	-	-	-	-
	T1	241	64	177	0.39	−0.50	1.28	0.39	1.29	−1.24	3.82	0.32	0.41	−0.38	−1.27	0.51	0.40	−1.32	−3.86	1.22	0.31	0.39
	T2	258	67	191	0.04	−0.77	0.85	0.92	−0.50	−2.22	1.22	0.57	0.69	−0.03	−0.84	0.78	0.95	0.51	−1.21	2.23	0.56	0.70
	T3	249	72	177	−0.43	−1.23	0.38	0.30	−1.44	−3.28	0.40	0.13	0.44	0.44	−0.37	1.24	0.29	1.42	−0.42	3.27	0.13	0.46
Added sugar	0	554	169	385	ref	-	-	-	ref	-	-	-	-	ref	-	-	-	ref	-	-	-	-
	T1	331	103	228	0.68	−0.02	1.38	0.06	0.20	−1.50	1.89	0.82	0.65	−0.67	−1.37	0.03	0.06	−0.19	−1.89	1.51	0.82	0.65
	T2	311	99	212	−0.53	−1.33	0.28	0.20	0.06	−1.32	1.45	0.93	0.23	0.53	−0.28	1.34	0.20	−0.03	−1.42	1.35	0.96	0.25
	T3	331	109	222	−0.30	−1.18	0.58	0.51	−0.40	−1.75	0.96	0.57	0.77	0.31	−0.57	1.19	0.49	0.42	−0.94	1.77	0.55	0.79

* Exposure amount was estimated as the proportion of daily intake with respect to ADI and was categorized into tertiles (T1–T3), with the no-intake group serving as a reference. The models were adjusted for age, amount of exercise, sleep quality, total energy intake, and parental education time. ** *p* value reflects the difference between boys and girls.

**Table 4:** Associations of sweetener consumption with fat mass and fat free mass according to Tanner stage.

Sweetener	Tanner Stage	*n*			Fat Mass %									Fat-Free Mass %								
			*n*	*n*	I and II				III–V				*p* **	I and II				III–V				*p* **
	Exposure Amount *		I and II	III–V	β	95% CI		*p*	β	95% CI		*p*		β	95% CI		*p*	β	95% CI		*p*	
Acesulfame-K	0	781	694	87	ref	-	-	-	ref	-	-	-	-	ref	-	-	-	ref	-	-	-	-
	T1	122	106	16	0.33	−0.98	1.64	0.62	−0.65	−3.77	2.48	0.69	0.67	−0.34	−1.65	0.97	0.61	0.64	−2.47	3.76	0.69	0.66
	T2	102	86	16	−0.32	−1.49	0.86	0.60	−3.17	−5.86	−0.47	0.02	0.69	0.32	−0.86	1.49	0.60	3.18	0.49	5.86	0.02	0.66
	T3	110	94	16	0.43	−0.72	1.58	0.46	−2.00	−5.32	1.33	0.24	0.13	−0.44	−1.59	0.71	0.45	1.97	−1.35	5.28	0.25	0.14
Aspartame	0	696	620	76	ref	-	-	-	ref	-	-	-	-	ref	-	-	-	ref	-	-	-	-
	T1	168	132	36	0.06	−0.90	1.01	0.91	−1.63	−3.51	0.25	0.09	0.58	−0.06	−1.02	0.89	0.90	1.58	−0.30	3.45	0.10	0.62
	T2	161	134	27	−1.03	−2.03	−0.03	0.04	−2.51	−4.75	−0.27	0.03	0.94	1.05	0.05	2.05	0.04	2.50	0.27	4.73	0.03	0.93
	T3	154	138	16	−0.94	−1.85	−0.03	0.04	−3.69	−6.54	−0.85	0.01	0.78	0.93	0.02	1.84	0.05	3.67	0.83	6.51	0.01	0.77
Sucralose	0	652	575	77	ref	-	-	-	ref	-	-	-	-	ref	-	-	-	ref	-	-	-	-
	T1	200	169	31	−0.30	−1.21	0.60	0.51	1.27	−0.81	3.35	0.23	0.15	0.33	−0.58	1.24	0.48	−1.23	−3.30	0.84	0.25	0.15
	T2	161	144	17	−0.76	−1.64	0.13	0.09	−3.48	−6.25	−0.72	0.01	0.10	0.74	−0.14	1.63	0.10	3.46	0.71	6.21	0.01	0.11
	T3	169	143	26	−0.07	−0.98	0.84	0.88	−1.78	−3.88	0.33	0.10	0.46	0.08	−0.83	0.99	0.87	1.79	−0.31	3.88	0.10	0.44
Glycyrrhizin	0	708	622	86	ref	-	-	-	ref	-	-	-	-	ref	-	-	-	ref	-	-	-	-
	T1	165	142	23	−0.76	−1.70	0.17	0.11	−1.43	−3.57	0.72	0.19	0.50	0.78	−0.16	1.72	0.10	1.41	−0.73	3.55	0.20	0.50
	T2	147	131	16	−0.20	−1.07	0.67	0.65	0.43	−2.08	2.93	0.74	0.77	0.22	−0.65	1.09	0.62	−0.38	−2.87	2.12	0.77	0.77
	T3	140	127	13	−1.00	−1.88	−0.12	0.03	−1.61	−4.16	0.95	0.22	0.19	1.00	0.12	1.88	0.03	1.63	−0.91	4.18	0.21	0.18
Stevioside	0	890	796	94	ref	-	-	-	ref	-	-	-	-	ref	-	-	-	ref	-	-	-	-
	T1	62	54	8	0.70	−0.57	1.97	0.28	−1.74	−6.83	3.34	0.50	0.47	−0.74	−2.01	0.54	0.26	1.66	−3.41	6.73	0.52	0.45
	T2	63	53	10	−0.55	−1.88	0.78	0.42	−4.03	−8.13	0.06	0.05	0.14	0.57	−0.77	1.90	0.40	4.01	−0.07	8.09	0.06	0.14
	T3	61	51	10	−1.30	−2.91	0.31	0.11	−0.09	−3.34	3.15	0.96	0.29	1.27	−0.35	2.88	0.12	−0.03	−3.27	3.21	0.99	0.27
Sorbitol	0	593	521	72	ref	-	-	-	ref	-	-	-	-	ref	-	-	-	ref	-	-	-	-
	T1	199	169	30	1.21	0.16	2.26	0.02	−0.63	−3.23	1.97	0.63	0.25	−1.22	−2.28	−0.17	0.02	0.66	−1.93	3.26	0.62	0.26
	T2	193	173	20	−0.05	−0.93	0.83	0.91	−0.29	−2.71	2.13	0.82	0.94	0.06	−0.82	0.94	0.89	0.30	−2.11	2.71	0.81	0.94
	T3	201	171	30	−0.62	−1.52	0.29	0.18	−2.39	−4.80	0.02	0.05	0.19	0.62	−0.29	1.53	0.18	2.42	0.01	4.82	0.05	0.17
Added sugar	0	496	441	55	ref	-	-	-	ref	-	-	-	-	ref	-	-	-	ref	-	-	-	-
	T1	272	227	45	0.53	−0.26	1.32	0.19	−0.19	−2.09	1.72	0.85	0.75	−0.53	−1.32	0.26	0.19	0.21	−1.69	2.11	0.83	0.74
	T2	240	200	40	−0.05	−0.88	0.78	0.90	−1.84	−3.68	0.01	0.05	0.17	0.07	−0.77	0.90	0.87	1.86	0.02	3.70	0.05	0.18
	T3	249	216	33	−0.09	−0.96	0.78	0.84	−0.88	−2.88	1.11	0.39	0.79	0.12	−0.75	0.99	0.79	0.88	−1.11	2.87	0.39	0.82

* Exposure amount was estimated as the proportion of daily intake with respect to ADI and was categorized into tertiles (T1–T3), with the no-intake group serving as a reference. The models were adjusted for age, sex, amount of exercise, sleep quality, total energy intake, and parental education time. ** *p* value reflects the difference between early and late Tanner stages.

## Data Availability

The data underlying in the current study are available from the corresponding author upon reasonable request.

## Supplementary Materials

The following are available online at https://www.mdpi.com/article/10.3390/nu15102319/s1, Table S1. Associations between urinary concentrations of three sweeteners and body composition; Table S2. Baseline characteristics of sweetener consumption; Table S3. Associations of sweetener consumption with fat mass and fat-free mass among girls according to Tanner stage; Table S4. Associations of sweetener consumption with fat mass and fat-free mass among boys according to Tanner stage; Table S5. Associations of sweetener consumption with fat mass and fat-free mass by obesity group.
