# Supplementary material for: Effects of Nonnutritive Sweeteners on Body Composition Changes during Pubertal Growth
Source: Nutrients. 2023 May 15;15(10):2319. doi: 10.3390/nu15102319 (PMC10224528; doi:10.3390/nu15102319)
Supplement: Supplementary file 1 [file nutrients-15-02319-s001.zip › nutrients-2261830-supplementary.pdf]

Online Supplementary Materials

Online Supplementary Materials 1

Table S1. Associations between urinary concentrations of three sweeteners and body composition (n = 455) 2

Table S2. Baseline characteristics of the sweetener consumption 3

Table S3. Associations of sweetener consumption with fat mass and fat-free mass among girls, by Tanner stage (n = 1239) 4

Table S4. Associations of sweetener consumption with fat mass and fat-free mass among boys, by Tanner stage (n = 654) 6

Table S5. Associations of sweetener consumption with fat mass and fat-free mass, by obesity group (n = 1893) 8

Table S1. Associations between urinary concentrations of three sweeteners and body composition (n = 455)

Sweetener	Exposure amount*	BMI-z score				Fat mass %				Fat-free mass %				Waist-to-height ratio			
		β	95% CI		P value	β	95% CI		P value	β	95% CI		P value	β	95% CI		P value
**Acesulfame-K**	0	ref	-	-	-	ref	-	-	-	ref	-	-	-	ref	-	-	-
	T1	0.02	−0.44	0.47	0.95	0.05	−3.11	3.22	0.97	−0.02	−3.18	3.15	0.99	-<0.01	−0.03	0.02	0.90
	T2	-<0.01	−0.44	0.43	1.00	−0.29	−3.40	2.82	0.86	0.29	−2.82	3.40	0.85	<0.01	−0.02	0.03	0.79
	T3	0.17	−0.25	0.60	0.42	1.15	−1.88	4.17	0.46	−1.14	−4.17	1.88	0.46	0.01	−0.02	0.03	0.48
**Sucralose**	0	ref	-	-	-	ref	-	-	-	ref	-	-	-	ref	-	-	-
	T1	−0.30	−0.67	0.07	0.11	−3.00	−5.59	−0.41	**0.02**	3.02	0.43	5.61	**0.02**	−0.01	−0.03	0.01	0.42
	T2	−0.06	−0.40	0.28	0.74	−0.30	−2.71	2.11	0.81	0.32	−2.09	2.73	0.79	0.01	−0.01	0.03	0.22
	T3	−0.08	−0.42	0.25	0.62	−0.17	−2.47	2.13	0.88	0.19	−2.10	2.49	0.87	−0.01	−0.03	0.01	0.30
**Stevioside**	0	ref	-	-	-	ref	-	-	-	ref	-	-	-	ref	-	-	-
	T1	0.02	−0.38	0.42	0.91	0.22	−2.61	3.04	0.88	−0.23	−3.05	2.59	0.87	<0.01	−0.02	0.03	0.78
	T2	0.23	−0.12	0.58	0.19	1.14	−1.30	3.58	0.36	−1.13	−3.57	1.31	0.36	0.01	−0.01	0.03	0.44
	T3	−0.30	−0.66	0.05	0.10	−2.23	−4.76	0.29	0.08	2.23	−0.29	4.76	0.08	−0.01	−0.04	0.01	0.16

*Exposure amount was estimated as the proportion of daily intake to ADI and was categorized into tertiles (T1–T3), with the no intake group as reference. The models were adjusted for age, sex, amount of exercise, sleep quality, total energy intake, and parental education time.

Table S2. Baseline characteristics of the sweetener consumption

Sweetener	Exposure amount*	Girls		Boys		P value **
		Mean	SD	Mean	SD	
**Acesulfame-K**	T1	0.12	0.08	0.17	0.13	0.01
	T2	0.7	0.39	1.03	0.56	<0.01
	T3	4.47	5.39	9.06	12.14	0.01
**Aspartame**	T1	0.09	0.05	0.11	0.05	0.08
	T2	0.32	0.15	0.39	0.21	0.41
	T3	3.94	6.97	8.92	16.94	0.01
**Sucralose**	T1	0.18	0.1	0.18	0.12	0.12
	T2	0.8	0.51	1.06	0.68	0.03
	T3	7.36	8.98	12.95	13.84	0.01
**Glycyrrhizin**	T1	0.02	0.01	0.02	0.02	0.03
	T2	0.07	0.03	0.08	0.04	0.05
	T3	0.33	0.33	0.33	0.37	0.13
**Stevioside**	T1	0.08	0.03	0.09	0.04	0.05
	T2	0.18	0.09	0.25	0.1	0.02
	T3	1.09	1.36	2.61	3.18	0.06
**Sorbitol**	T1	17.46	12.61	21.2	12.14	0.09
	T2	121.61	59.5	161.11	80.95	0.04
	T3	615.7	544.74	1077.69	1744.98	0.04
**Added sugar**	T1	1.05	0.7	1.45	1.13	0.02
	T2	4.11	1.74	5.28	2.38	0.06
	T3	18.58	15.43	23.2	15.32	<0.01

*Exposure amount was estimated as the proportion of daily intake to ADI and was categorized into tertiles (T1–T3), with the no intake group as reference. The models were adjusted for age, amount of exercise, sleep quality, total energy intake, and parental education time. **P value reflects the difference between the early and late stages of pubertal growth

Table S3. Associations of sweetener consumption with fat mass and fat-free mass among girls, by Tanner stage (n = 1239)

Sweetener	Tanner stage	Fat mass %									Fat-free mass %									
		I & II				III–V				P value **	I & II				III–V				P value **
	Exposure amount*	β	95% CI		P value	β	95% CI		P value		β	95% CI		P value	β	95% CI		P value	
	0	ref	-	-	-	ref	-	-	-	-	ref	-	-	-	ref	-	-	-	-
**Acesulfame-K**	T1	0.77	−0.60	2.14	0.27	−1.11	−4.23	2.01	0.49	0.46	−0.78	−2.16	0.59	0.26	1.11	−2.00	4.22	0.49	0.46
	T2	−0.51	−1.67	0.65	0.39	−3.10	-6.21	0.02	0.05	0.84	0.52	−0.65	1.68	0.39	3.09	−0.01	6.20	0.05	0.80
	T3	−0.76	−2.26	0.73	0.32	−3.13	-6.62	0.36	0.08	0.87	0.74	−0.76	2.24	0.33	3.08	−0.39	6.56	0.08	0.88
**Aspartame**	0	ref	-	-	-	ref	-	-	-	-	ref	-	-	-	ref	-	-	-	-
	T1	−0.41	−1.37	0.55	0.40	−2.28	−4.21	−0.36	**0.02**	0.30	0.39	−0.57	1.35	0.43	2.23	0.31	4.14	**0.02**	0.32
	T2	−1.12	−2.22	−0.02	**0.05**	−3.15	−5.53	−0.77	**0.01**	0.84	1.15	0.05	2.25	**0.04**	3.17	0.80	5.54	**0.01**	0.83
	T3	−1.34	−2.39	−0.29	**0.01**	−2.99	−5.88	−0.11	**0.04**	0.93	1.32	0.27	2.38	**0.01**	2.98	0.10	5.86	**0.04**	0.90
**Sucralose**	0	ref	-	-	-	ref	-	-	-	-	ref	-	-	-	ref	-	-	-	-
	T1	−0.73	−1.70	0.25	0.14	3.13	0.43	5.83	**0.02**	**0.02**	0.76	−0.22	1.73	0.13	−3.10	−5.80	−0.40	**0.03**	**0.02**
	T2	−0.21	−1.23	0.80	0.68	−2.28	−5.06	0.49	0.11	0.44	0.21	−0.81	1.23	0.68	2.26	−0.51	5.03	0.11	0.47
	T3	−0.63	−1.63	0.38	0.22	−0.84	−3.31	1.63	0.50	0.66	0.63	−0.38	1.64	0.22	0.85	−1.61	3.32	0.50	0.67
**Glycyrrhizin**	0	ref	-	-	-	ref	-	-	-	-	ref	-	-	-	ref	-	-	-	-
	T1	−0.44	−1.31	0.43	0.32	−1.86	−3.91	0.19	0.08	0.59	0.47	−0.40	1.34	0.29	1.85	−0.20	3.89	0.08	0.58
	T2	−0.54	−1.35	0.28	0.20	−0.40	−2.79	2.00	0.75	0.34	0.57	−0.24	1.39	0.17	0.45	−1.94	2.85	0.71	0.34
	T3	−0.65	−1.56	0.27	0.17	−2.31	−4.78	0.16	0.07	**0.03**	0.68	−0.24	1.60	0.15	2.34	−0.12	4.81	0.06	**0.03**
**Stevioside**	0	ref	-	-	-	ref	-	-	-	-	ref	-	-	-	ref	-	-	-	-
	T1	0.09	−1.27	1.44	0.90	−1.09	-6.31	4.14	0.68	0.24	−0.12	−1.47	1.24	0.87	−1.09	-6.31	4.14	0.68	0.22
	T2	−1.23	−2.67	0.20	0.09	−2.30	-6.62	2.01	0.30	0.35	1.22	−0.22	2.66	0.10	−2.30	-6.62	2.01	0.30	0.35
	T3	−0.16	−1.90	1.57	0.85	0.17	−3.05	3.39	0.92	0.46	0.13	−1.61	1.87	0.88	0.17	−3.05	3.39	0.92	0.43
**Sorbitol**	0	ref	-	-	-	ref	-	-	-	-	ref	-	-	-	ref	-	-	-	-
	T1	0.82	−0.14	1.77	0.09	−0.44	−3.04	2.17	0.74	0.12	−0.82	−1.78	0.14	0.10	0.48	−2.12	3.08	0.72	0.12
	T2	0.20	−0.67	1.08	0.65	−0.38	−2.81	2.05	0.76	0.52	−0.19	−1.07	0.69	0.67	0.40	−2.02	2.82	0.74	0.51
	T3	−0.11	−0.98	0.75	0.80	−2.37	−4.84	0.10	0.06	0.35	0.13	−0.74	1.00	0.77	2.42	−0.04	4.88	0.06	0.31
**Added sugar**	0	ref	-	-	-	ref	-	-	-	-	ref	-	-	-	ref	-	-	-	-

T1	0.32	−0.43	1.07	0.40	0.31	−1.75	2.38	0.77	0.42	−0.32	−1.07	0.43	0.41	−0.29	−2.35	1.77	0.78	0.42
T2	−0.39	−1.29	0.51	0.40	−1.57	−3.79	0.65	0.17	0.31	0.39	−0.51	1.30	0.39	1.60	−0.62	3.81	0.16	0.32
T3	−0.50	−1.50	0.51	0.33	−0.38	−2.62	1.86	0.74	0.73	0.52	−0.49	1.53	0.31	0.38	−1.85	2.62	0.74	0.69

*Exposure amount was estimated as the proportion of daily intake to ADI and was categorized into tertiles (T1–T3), with the no intake group as reference. The models were adjusted for age, amount of exercise, sleep quality, total energy intake, and parental education time. **P value reflects the difference between the early and late stages of pubertal growth.

Table S4. Associations of sweetener consumption with fat mass and fat-free mass among boys, by Tanner stage (n = 654)

Sweetener		Fat mass %									Fat-free mass %								
	Tanner stage	I & II				III–V				P value **	I & II				III–V				P value **
	Exposure amount*	β	95% CI		P value	β	95% CI		P value		β	95% CI		P value	β	95% CI		P value	
Acesulfame-K	0	ref	-	-	-	ref	-	-	-	-	ref	-	-	-	ref	-	-	-	-
	T1	0.32	−2.12	2.76	0.80	0.57	−3.92	5.06	0.80	0.84	−0.34	−2.79	2.11	0.79	−0.63	−5.10	3.85	0.78	0.83
	T2	−0.86	−2.29	0.58	0.24	0.77	−3.47	5.01	0.72	0.99	0.86	−0.58	2.30	0.24	−0.66	−4.89	3.58	0.76	0.99
	T3	1.01	−0.36	2.38	0.15	−1.73	−5.95	2.49	0.42	0.36	−1.02	−2.40	0.36	0.15	1.69	−2.52	5.89	0.44	0.37
Aspartame	0	ref	-	-	-	ref	-	-	-	-	ref	-	-	-	ref	-	-	-	-
	T1	−0.84	−2.07	0.39	0.18	−1.89	−4.18	0.40	0.11	0.38	0.84	−0.39	2.08	0.18	1.79	−0.49	4.08	0.13	0.39
	T2	−1.88	−3.48	−0.28	**0.02**	−3.33	−5.99	−0.68	**0.02**	0.53	1.90	0.29	3.50	**0.02**	3.37	0.71	6.02	**0.02**	0.51
	T3	−1.19	−2.27	−0.11	**0.03**	−1.71	−5.55	2.13	0.39	0.93	1.19	0.11	2.27	**0.03**	1.67	−2.17	5.51	0.40	0.95
Sucralose	0	ref	-	-	-	ref	-	-	-	-	ref	-	-	-	ref	-	-	-	-
	T1	−1.73	−3.17	−0.30	**0.02**	1.91	−1.30	5.13	0.25	0.59	1.73	0.29	3.18	**0.02**	−1.84	−5.04	1.36	0.26	0.58
	T2	−1.07	−2.26	0.12	0.08	−1.07	−4.93	2.79	0.59	0.07	1.04	−0.16	2.23	0.09	1.05	−2.80	4.90	0.59	0.07
	T3	0.02	−0.96	1.01	0.96	−1.63	−4.82	1.55	0.32	0.49	−0.01	−1.00	0.98	0.99	1.71	−1.46	4.89	0.29	0.48
Glycyrrhizin	0	ref	-	-	-	ref	-	-	-	-	ref	-	-	-	ref	-	-	-	-
	T1	−1.62	−2.87	−0.36	**0.01**	−0.81	−4.52	2.91	0.67	**0.03**	1.65	0.39	2.90	**0.01**	0.77	−2.92	4.47	0.68	**0.03**
	T2	−0.69	−1.75	0.37	0.20	−0.26	-11.68	11.16	0.96	0.25	0.73	−0.34	1.80	0.18	0.26	-11.21	11.72	0.97	0.24
	T3	−0.37	−1.60	0.86	0.56	−5.42	-10.34	−0.50	**0.03**	0.40	0.39	−0.85	1.63	0.54	5.50	0.60	10.39	**0.03**	0.40
Stevioside	0	ref	-	-	-	ref	-	-	-	-	ref	-	-	-	ref	-	-	-	-
	T1	−0.01	−1.46	1.45	0.99	12.38	1.46	23.30	**0.03**	0.99	−0.02	−1.48	1.43	0.98	12.38	1.46	23.30	**0.03**	0.98
	T2	−0.88	−2.42	0.66	0.26	−1.85	-6.11	2.42	0.40	0.10	0.93	−0.61	2.48	0.24	−1.85	-6.11	2.42	0.40	0.10
	T3	−1.13	−2.88	0.62	0.21	5.69	-13.83	25.21	0.57	0.99	1.11	−0.65	2.87	0.22	5.69	-13.83	25.21	0.57	0.99
Sorbitol	0	ref	-	-	-	ref	-	-	-	-	ref	-	-	-	ref	-	-	-	-
	T1	0.70	−0.64	2.04	0.31	−0.54	−5.34	4.26	0.83	0.18	−0.68	−2.03	0.67	0.33	0.54	−4.26	5.34	0.83	0.18
	T2	0.43	−0.69	1.54	0.46	1.83	−2.09	5.76	0.36	0.45	−0.38	−1.51	0.74	0.50	−1.75	−5.68	2.17	0.38	0.45
	T3	−1.58	−2.86	−0.30	**0.02**	−2.69	-6.60	1.21	0.18	0.43	1.59	0.30	2.87	**0.02**	2.65	−1.25	6.55	0.19	0.42

**Added sugar**	0	ref	-	-	-	ref	-	-	-	-	ref	-	-	-	ref	-	-	-	-
	T1	0.66	−0.41	1.73	0.23	0.07	−3.52	3.67	0.97	0.06	−0.64	−1.72	0.44	0.24	−0.09	−3.68	3.49	0.96	0.06
	T2	−0.37	−1.48	0.74	0.52	−1.15	−5.17	2.87	0.58	0.24	0.39	−0.72	1.50	0.49	1.17	−2.84	5.19	0.57	0.25
	T3	−0.15	−1.16	0.87	0.78	1.65	−2.67	5.96	0.46	0.41	0.18	−0.83	1.20	0.72	−1.62	−5.92	2.68	0.46	0.41

*Exposure amount was estimated as the proportion of daily intake to ADI and was categorized into tertiles (T1–T3), with the no intake group as reference. The models were adjusted for age, amount of exercise, sleep quality, total energy intake, and parental education time. **P value reflects the difference between early and late Tanner stages.

Table S5. Associations of sweetener consumption with fat mass and fat-free mass, by obesity group (n = 1893)

Sweetener	Obesity group	Fat mass %									Fat-free mass %								
		Obesity				Normal				P value **	Obesity				Normal				P value **
	Exposure amount*	β	95% CI		P value	β	95% CI		P value		β	95% CI		P value	β	95% CI		P value	
**Acesulfame-K**	0	ref	-	-	-	ref	-	-	-	-	ref	-	-	-	ref	-	-	-	-
	T1	−1.72	−5.98	2.54	0.43	0.60	−0.41	1.62	0.24	0.90	1.69	−2.57	5.95	0.44	−0.61	−1.62	0.41	0.24	0.90
	T2	1.36	−3.50	6.22	0.58	−0.95	−1.88	−0.02	**0.05**	0.24	−1.40	-6.26	3.47	0.57	0.96	0.03	1.89	**0.04**	0.24
	T3	0.33	−3.46	4.11	0.87	0.11	−0.86	1.08	0.82	0.96	−0.37	−4.15	3.42	0.85	−0.12	−1.09	0.85	0.81	0.94
**Aspartame**	0	ref	-	-	-	ref	-	-	-	-	ref	-	-	-	ref	-	-	-	-
	T1	−0.26	−2.81	2.30	0.84	0.26	−0.51	1.03	0.50	0.74	0.26	−2.29	2.82	0.84	−0.28	−1.05	0.49	0.48	0.76
	T2	1.18	−2.03	4.40	0.47	−0.92	−1.72	−0.12	**0.02**	**<0.01**	−1.16	−4.38	2.05	0.48	0.93	0.13	1.73	**0.02**	**<0.01**
	T3	−2.14	−4.94	0.66	0.14	−0.90	−1.68	−0.11	**0.03**	0.08	2.14	−0.66	4.94	0.14	0.88	0.09	1.67	**0.03**	0.09
**Sucralose**	0	ref	-	-	-	ref	-	-	-	-	ref	-	-	-	ref	-	-	-	-
	T1	1.22	−1.36	3.80	0.35	0.20	−0.56	0.95	0.60	0.93	−1.23	−3.81	1.35	0.35	−0.18	−0.93	0.58	0.65	0.95
	T2	0.68	−2.53	3.88	0.68	−0.99	−1.75	−0.23	**0.01**	0.22	−0.72	−3.93	2.49	0.66	0.98	0.22	1.74	**0.01**	0.22
	T3	−0.26	−3.25	2.73	0.87	−0.56	−1.31	0.20	0.15	0.06	0.23	−2.76	3.22	0.88	0.56	−0.19	1.32	0.14	0.06
**Glycyrrhizin**	0	ref	-	-	-	ref	-	-	-	-	ref	-	-	-	ref	-	-	-	-
	T1	−2.78	−5.31	−0.24	**0.03**	−0.22	−1.01	0.56	0.58	**<0.01**	2.78	0.25	5.31	**0.03**	0.23	−0.56	1.01	0.57	**<0.01**
	T2	1.70	−1.21	4.61	0.25	−0.17	−0.91	0.58	0.66	0.09	−1.69	−4.60	1.22	0.26	0.19	−0.56	0.93	0.63	0.09
	T3	−0.61	−3.95	2.73	0.72	−1.18	−1.91	−0.45	**<0.01**	**<0.01**	0.60	−2.74	3.94	0.73	1.18	0.46	1.91	**<0.01**	**<0.01**
**Stevioside**	0	ref	-	-	-	ref	-	-	-	-	ref	-	-	-	ref	-	-	-	-
	T1	−0.19	−4.27	3.88	0.93	0.53	−0.58	1.64	0.35	0.28	0.25	−3.83	4.33	0.90	0.53	−0.58	1.64	0.35	0.26
	T2	−0.08	−4.64	4.48	0.97	−0.94	−2.12	0.24	0.12	0.54	0.04	−4.52	4.60	0.99	−0.94	−2.12	0.24	0.12	0.55
	T3	−4.43	-11.43	2.56	0.22	−0.48	−1.70	0.75	0.44	0.19	4.42	−2.58	11.42	0.22	−0.48	−1.70	0.75	0.44	0.20
**Sorbitol**	0	ref	-	-	-	ref	-	-	-	-	ref	-	-	-	ref	-	-	-	-

	T1	2.35	−0.14	4.83	0.07	0.05	−0.81	0.91	0.91	0.28	−2.36	−4.84	0.13	0.06	−0.05	−0.91	0.81	0.91	0.28
	T2	1.42	−1.35	4.20	0.32	−0.18	−0.90	0.54	0.62	0.37	−1.45	−4.23	1.32	0.31	0.20	−0.52	0.91	0.59	0.35
	T3	0.22	−2.62	3.06	0.88	−0.88	−1.61	−0.15	**0.02**	**<0.01**	−0.22	−3.06	2.62	0.88	0.88	0.15	1.61	**0.02**	**<0.01**
**Added sugar**	0	ref	-	-	-	ref	-	-	-	-	ref	-	-	-	ref	-	-	-	-
	T1	1.40	−1.05	3.84	0.26	0.51	−0.14	1.16	0.13	0.10	−1.43	−3.88	1.01	0.25	−0.50	−1.15	0.15	0.14	0.09
	T2	0.50	−2.31	3.31	0.73	−0.29	−0.95	0.37	0.38	0.85	−0.52	−3.33	2.29	0.72	0.31	−0.35	0.97	0.36	0.87
	T3	−0.84	−3.60	1.93	0.55	−0.51	−1.20	0.17	0.14	0.55	0.81	−1.96	3.58	0.57	0.53	−0.16	1.21	0.13	0.55

*Exposure amount was estimated as the proportion of daily intake to ADI and was categorized into tertiles (T1–T3), with the no intake group as reference. The models were adjusted for age, amount of exercise, sleep quality, total energy intake, and parental education time. **P value reflects the difference between obesity and nonobesity.
